# Utility of Recent Studies to Assess the National Research Council 2001 Estimates of Cancer Risk from Ingested Arsenic

**DOI:** 10.1289/ehp.1002427

**Published:** 2010-10-28

**Authors:** Herman Gibb, Cary Haver, David Gaylor, Santhini Ramasamy, Janice S. Lee, Danelle Lobdell, Timothy Wade, Chao Chen, Paul White, Reeder Sams

**Affiliations:** 1 Tetra Tech Sciences, Arlington, Virginia, USA; 2 Gaylor and Associates, LLC, Eureka Springs, Arkansas, USA; 3 Office of Water, U.S. Environmental Protection Agency, Washington, DC, USA; 4 National Center for Environmental Assessment and; 5 National Health and Environmental Effects Research Laboratory, U.S. Environmental Protection Agency, Research Triangle Park, NC; 6 National Center for Environmental Assessment, U.S. Environmental Protection Agency, Washington, DC, USA

**Keywords:** arsenic, bladder, cancer, dose response, drinking water, lung, risk assessment

## Abstract

**Objective:**

The purpose of this review is to evaluate the impact of recent epidemiologic literature on the National Research Council (NRC) assessment of the lung and bladder cancer risks from ingesting low concentrations (< 100 μg/L) of arsenic-contaminated water.

**Data sources, extraction, and synthesis:**

PubMed was searched for epidemiologic studies pertinent to the lung and bladder cancer risk estimates from low-dose arsenic exposure. Articles published from 2001, the date of the NRC assessment, through September 2010 were included. Fourteen epidemiologic studies on lung and bladder cancer risk were identified as potentially useful for the analysis.

**Conclusions:**

Recent epidemiologic studies that have investigated the risk of lung and bladder cancer from low arsenic exposure are limited in their ability to detect the NRC estimates of excess risk because of sample size and less than lifetime exposure. Although the ecologic nature of the Taiwanese studies on which the NRC estimates are based present certain limitations, the data from these studies have particular strengths in that they describe lung and bladder cancer risks resulting from lifetime exposure in a large population and remain the best data on which to conduct quantitative risk assessment. Continued follow-up of a population in northeastern Taiwan, however, offers the best opportunity to improve the cancer risk assessment for arsenic in drinking water. Future studies of arsenic < 100 μg/L in drinking water and lung and bladder cancer should consider adequacy of the sample size, the synergistic relationship of arsenic and smoking, duration of arsenic exposure, age when exposure began and ended, and histologic subtype.

In 1996, the U.S. Environmental Protection Agency (U.S. EPA) Office of Water requested the National Research Council (NRC) to independently review the available scientific database for the health effects of arsenic in drinking water and to evaluate the validity of the 1988 assessment. The result was the NRC report *Arsenic in Drinking Water* (NRC 1999). The report analyzed bladder cancer risks from arsenic ingestion using mortality data from a southwest Taiwanese population (Chen et al. 1992; Wu et al. 1989).

In January 2001, using the newly available data, the U.S. EPA issued a maximum contaminant level (MCL) of 10 μg/L for arsenic (U.S. EPA 2001a). The 23 January 2006 compliance date for the arsenic standard gave the U.S. EPA time to reassess the scientific and cost issues and to seek further public input on the arsenic regulation (U.S. EPA 2001b). As part of this re-evaluation, the NRC was requested to update its 1999 report. The resulting report, *Arsenic in Drinking Water—2001 Update* (NRC 2001), concluded that there was a sound database on the carcinogenic effects of arsenic in humans that was adequate for the purpose of risk assessment, and that lung and bladder cancer should continue to be the focus of arsenic risk assessment for regulatory decision making. The NRC report also concluded that the human data from southwestern Taiwan (Chen et al. 1988a, 1992; Wu et al. 1989) used by the U.S. EPA (2001a) in its risk assessment remained the most appropriate data to determine lifetime cancer risk estimates. Table 1 contains the NRC estimates of excess lifetime lung and bladder cancer risks.

The purpose of this review is to evaluate whether the recent literature on arsenic and lung and bladder cancer is consistent with or contradicts the NRC (2001) with respect to cancer risk at low (≤ 100 μg arsenic/L) concentrations of arsenic in drinking water. We searched PubMed for all epidemiology studies on arsenic in drinking water and lung and bladder cancer published from 2001 through September 2010. The following search terms were included: arsenic, water, cancer, lung or bladder, and epidemiology. As of September 2010, 195 studies and articles were identified in a PubMed search. Studies considered in this review specifically assessed lung and bladder cancer outcomes and exposure to low levels (≤ 100 μg/L) of arsenic in drinking water. Studies not assessing lung and bladder cancer from low-arsenic exposure and studies not specific to exposure from drinking water were excluded. Fourteen epidemiologic studies that examined the risk of lung and bladder cancer from low arsenic concentrations (≤ 100 μg/L) in drinking water were identified (Baastrup et al. 2008; Bates et al. 2004; Chen et al. 2004, 2010a, 2010b; Han et al. 2009; Heck et al. 2009; Karagas et al. 2004; Lamm et al. 2004; Meliker et al. 2007, 2010; Michaud et al. 2004; Mostafa et al. 2008; Steinmaus et al. 2003).

## Ability of an Epidemiology Study to Detect the Excess Risks Estimated by the NRC

The lung and bladder cancer risks estimated by the NRC (2001) were based on an ecological study of a population in an arsenic-endemic area of southwest Taiwan. The study was unique in many aspects. Exposure began early in the 20th century, and the population was extremely stable (Wu et al. 1989). Chen et al. (1992) commented that the affected population lived in a confined area and shared similar socioeconomic status, living environments, lifestyles, dietary patterns, and even medical facilities; the only major difference in environmental exposures in the population appeared to be the differences in arsenic concentrations in drinking water. Assuming that the effect of arsenic is additive to the background risk, risks of arsenic-induced lung and, in particular, bladder cancer would be easier to detect in Taiwan than in the United States because of lower background risks in Taiwan. The age-adjusted bladder cancer incidence is four times lower in southeastern Asia than in North America; the age-adjusted lung cancer incidence is about 40% lower among males and about three times lower in females in southeastern Asia than in North America [International Agency for Research on Cancer (IARC) 2008].

Although many strengths existed for quantitative risk assessment, the study had some limitation given its ecologic design. The concentrations of arsenic in drinking water for those in the study were based on the median value for the wells in the village. The NRC recognized that use of the median value presented some uncertainty, but a sensitivity analysis suggested that use of the median value would present little effect on the risk estimate (NRC 2001). The NRC further commented that the use of an external comparison population minimized the effect of exposure misclassification on the risk assessment.

Arsenic exposure in the southwest Taiwan population was reported to have begun from about 1900 to 1910 (Tseng et al. 1968). The arsenic exposure therefore could be considered to be lifelong in the population that was the basis of the NRC cancer risk estimates. This is a particularly important consideration if the latency period from exposure to development of disease is long or if arsenic has different effects at different life stages. Although data on arsenic concentrations do not exist for each year that the population was exposed, Chen et al. (1988b) indicate that arsenic concentrations in the wells in the endemic area were stable over approximately a 13-year period, and it is reasonable to assume that the concentrations were stable over a longer period of time.

For several reasons, it would be difficult for epidemiologic studies to detect the NRC-estimated cancer risks in the United States. First, the excess lung and bladder cancer lifetime risks for low arsenic exposures are small compared with the background lifetime risks for these diseases. At 10 μg arsenic/L, the current U.S. EPA MCL for arsenic in drinking water (U.S. EPA 2001c), the excess risk for lung cancer would be 30- to 50-fold lower than the lifetime risk of lung cancer in the United States. The excess risk for bladder cancer would be approximately 8- to 14-fold lower than the lifetime risk of bladder cancer in the United States. The sex-specific lung and bladder cancer excess and relative risks (RRs) from lifetime exposure to 10 μg arsenic/L are compared in Table 2. The excess risk is the risk derived by the NRC for 10 μg arsenic/L; the RR is the excess risk plus the background risk in the United States [National Cancer Institute (NCI) 2010] divided by the background risk. The highest RR estimated from arsenic in drinking water is for bladder cancer among females, largely because the background risk of bladder cancer among U.S. females (0.0094) is lower than the background risk for lung cancer among females and the background risks of lung and bladder cancer among males. Age-standardized risks for male and female lung and bladder cancer follow a similar pattern worldwide (IARC 2007). Evidence that the RRs from arsenic exposure in the drinking water are highest for bladder cancer among females is supported by studies in Chile (Marshall et al. 2007) and Taiwan (Chen et al. 1985).

Second, the study population on which the NRC estimates are based is determined to have lifetime or near-lifetime exposure. Finding other populations with lifetime exposure would be difficult. Besides the shorter exposure duration experienced by those with less than lifetime exposure, exposures at some life stages (e.g., infant vs. adult) could present more risk than at other life stages (Halmes et al. 2000; Smith et al. 2006; Waalkes et al. 2003). Both of these exposure issues would diminish the ability of a study to detect the risks estimated by NRC.

Finally, dietary arsenic would play a much greater role in misclassification of exposure at low doses than it would at high doses (Cantor and Lubin 2007; Kile et al. 2007; Uchino et al. 2006). As the NRC noted, the detection of the theoretical risks that they estimated would require a large population consuming drinking water containing arsenic over an extended period of time.

In Table 3, we demonstrate the sample sizes needed to detect the bladder cancer risk from lifetime arsenic exposure in females based on the NRC estimates and a background lifetime bladder cancer risk among females in the United States from the Surveillance Epidemiology End Results (SEER) data (NCI 2010), assuming 80% statistical power and a type 1 error (false-positive rate) of *p* = 0.05. Sample sizes were estimated using STATA 10 (StataCorp LP, College Station, TX). The sample size needed to detect an excess bladder cancer risk in females is smaller than the sample size needed to detect a lung cancer risk in females or a lung or bladder cancer risk in males, as the RR for bladder cancer in females is greater (Table 2). Less than lifetime exposure and exposure misclassification will increase the sample sizes described in Table 3. Furthermore, if lung and bladder cancer risks exist at low doses, they could be overestimated by the NRC’s linear model of high-dose exposure. Thus, for several reasons, the sample sizes described in Table 3 are conservative, and the sample sizes required to detect arsenic-induced cancer risks could actually be much greater. The approach used for the sample size calculations presented in Table 3 is also applicable to unmatched case–control studies. It should be clear that sample size calculations are essentially approximate, and it is not practical to present more precise calculations under all conceivable conditions (e.g., matched vs. unmatched, discrete vs. continuous variables). Even under the same condition such as discrete (or categorized) variable, the sample size can depend on the number of categories involved. For instance, Mehta and Hilton (1993) have demonstrated that sample sizes calculated under conditional and nonconditional distributions could differ significantly, depending on the number of categories for the target variable involved.

## Epidemiology Studies Conducted Since the NRC Report

The epidemiologic studies that have examined the risk of lung and bladder cancer published since NRC (2001) include four cohort studies (Baastrup et al. 2008; Chen et al. 2004, 2010a, 2010b), seven case–control studies (Bates et al. 2004; Heck et al. 2009; Karagas et al. 2004; Meliker et al. 2010; Michaud et al. 2004; Mostafa et al. 2008; Steinmaus et al. 2003), and three ecologic studies (Han et al. 2009; Lamm et al. 2004; Meliker et al. 2007). Seven studies examined bladder cancer risk (Bates et al. 2004; Chen et al. 2010b; Karagas et al. 2004; Lamm et al. 2004; Meliker et al. 2010; Michaud et al. 2004, Steinmaus et al. 2003), four examined lung cancer risk (Chen et al. 2004, 2010a, Heck et al. 2009; Mostafa et al. 2008), and three examined both lung and bladder cancer risk (Baastrup et al. 2008; Han et al. 2009; Meliker et al. 2007). Of these 14 studies, 11 used arsenic concentration in drinking water as the measure of arsenic exposure (Baastrup et al. 2008; Bates et al. 2004; Chen et al. 2004, 2010a, 2010b; Han et al. 2009; Heck et al. 2009; Lamm et al. 2004; Meliker et al. 2007, 2010; Mostafa et al. 2008; Steinmaus et al. 2003). Three studies (Heck et al. 2009; Karagas et al. 2004; Michaud et al. 2004) examined toenail arsenic as a surrogate measure of exposure. Toenail arsenic is believed to represent primarily ingested arsenic. Table 4 briefly describes the 14 epidemiologic studies and indicates whether there is any evidence of significantly (*p* < 0.05) increased lung or bladder cancer risks associated with drinking water concentrations < 100 μg arsenic/L. Brief summaries of each study are provided below.

### Cohort studies

Baastrup et al. (2008) reported on 56,378 Danish men and women, 50–64 years of age at enrollment between 1993 and 1997, who were followed until date of first cancer diagnosis, emigration, death, or 1 August 2003. Time-weighted average exposure and cumulative exposure to arsenic were based on residential history between 1970 and 2003. Arsenic concentrations for utilities (i.e., drinking water treatment systems) were based on data for 1987 through 2004, with most measurements taken between 2002 and 2004. The average at each water utility was assumed to represent the arsenic concentrations throughout the study period of 1970–2003. Questionnaires administered to study participants assessed potential lifestyle, occupational, and environmental risks. During the follow-up period, 214 bladder cancer cases and 402 lung cancer cases were diagnosed. The time-weighted arsenic exposure of the cohort members ranged from 0.05 to 25.3 μg/L, with a median and mean concentration of 0.7 μg/L and 1.2 μg/L, respectively. Incidence rate ratios (IRRs), adjusted for a variety of different variables, found no association between the time-weighted average or cumulative exposures and lung or bladder cancer. Relative risks were not reported by sex.

In an earlier study, Chen et al. (2004) described 2,503 residents of southwest Taiwan and 8,088 residents of northeast Taiwan followed for an average period of 8 years. All those in the cohort had consumed arsenic-contaminated water for > 50 years. Those in the cohort from southwest Taiwan included a group of patients with blackfoot disease who were age, sex, and residentially matched with healthy community controls. Each study participant was administered a structured questionnaire to obtain sociodemographic, residential, and occupational history, well water–intake history, and cigarette and alcohol consumption information. Average arsenic concentration in drinking water was used as the exposure metric. RRs and 95% confidence intervals (CIs) were estimated by Cox proportional hazards models. Adjustment variables in the final model included age, sex, years of schooling, study cohort (blackfoot disease and matched controls; residents of the arsenic-endemic areas in southwest and northeast Taiwan), smoking status, and habitual alcohol consumption. The researchers identified 139 new, pathologically confirmed lung cancer cases. When compared with the referent group of < 10 μg/L, the adjusted RRs (95% CIs) for lung cancers were 1.09 (0.63–1.91); 2.28 (1.22–4.27); 3.03 (1.62–5.69); and 3.29 (1.60–6.78) for average arsenic concentrations of 10–99, 100–299, 300–699, and ≥ 700 μg/L, respectively. The trend was statistically significant. A strong synergism was found between arsenic in water and cigarette consumption. For those smoking ≥ 25 pack-years, RRs (95% CIs) for lung cancer were 3.8 (1.29–11.2), 5.93 (2.19–16.1), and 11.10 (3.32–37.2) for average arsenic concentrations of < 10, 10–699, and ≥ 700 μg/L, respectively.

In the Taiwan cohort, Chen et al. (2010a, 2010b) studied 8,086 northeastern Taiwanese residents. Drinking water concentration was reported at time of enrollment. A total of 3,901 well-water samples were collected from 4,584 (85.1%) households during the home interview. No information on the arsenic concentration of the well water at prior residences was obtained. Detailed residential history and corresponding well-water arsenic concentrations were used to calculate cumulative exposure as well as starting and ending age of exposure and duration of exposure. Chen et al. (2010a) studied various exposure metrics such as the effect of age when drinking water containing arsenic was started and stopped, years of drinking well water, and cumulative exposure in estimating lung cancer risk. RRs were adjusted for age, sex, education, cigarette-smoking status, and alcohol consumption at enrollment. The RRs and 95% CIs for 100–300 and > 300 μg arsenic/L when compared with < 10 μg arsenic/L were 1.54 (0.97–2.46) and 2.25 (1.43–3.55), respectively. There was no apparent increased risk at concentrations between 10 and 100 μg arsenic/L, but when duration of exposure was accounted for, all levels of exposure including low concentrations were in the direction of an increased risk of lung cancer. These associations tended to increase with longer durations of exposure. A synergistic effect of arsenic exposure and cigarette smoking was found for squamous- and small-cell carcinomas, but not for adenocarcinoma. Chen et al. (2010b) identified 45 incident cases of urinary cancer. Data showed a significant (*p* < 0.001) monotonic increasing risk of urinary cancer with arsenic concentration. Compared with those consuming < 10 μg arsenic/L, the age and sex-adjusted RRs (95% CIs) for 10–49.9, 50–99.9, 100–299.9, and ≥ 300 μg arsenic/L were 1.7 (0.56–5.19), 2.49 (0.73–8.59), 4.18 (1.3–12.8), and 7.73 (2.69–22.3), respectively. The trend was highly significant (*p* < 0.001). Urinary cancer RRs (95% CIs) for cumulative arsenic exposures 400–1,000, 1,000–5,000, 5,000–10,000, and ≥ 10,000 μg/L-year were 1.16 (0.29–4.64), 2.44 (0.91–6.5), 3.88 (1.18–12.7), and 7.55 (2.79–20.4), respectively, compared with < 400 μg/L-year. There was a monotonic increase in risk with cumulative exposure. The association with arsenic was strongest for urothelial cancer (transitional-cell carcinoma).

### Case–control studies

Bates et al. (2004) described 114 incident bladder cancer cases in Argentina from previously determined high- and medium-exposure counties matched on age, sex, and county with 114 controls. Cases were between the ages of 20 and 80, had lived in the high- and medium-exposure counties between 1996 and 2000, and were alive at the time of the study. Controls were identified from voter registration records. The following data were collected: residential history, smoking, consumption of fluids, and occupational and medical history. Water samples were collected at the current residence and all previous residences and analyzed for arsenic. Where the well at a former residence was closed, a sample was collected from a nearby (proxy) well. Exposure was defined by average arsenic concentration, time-weighted water arsenic consumption, which was adjusted for total fluid consumption, and consumption of well water during 10-year intervals before the interview. The investigation found no evidence of an association between measures of exposure, based on arsenic concentration, and bladder cancer. Drinking well water for 51 to 70 years before the interview was found to be associated with an increased risk of bladder cancer among ever-smokers [odds ratio (OR) = 2.5; 95% CI, 1.1–5.5) but not among never smokers. Mean, median, and range time-weighted arsenic exposure levels, respectively, were 20, 1.3, and 0 to 212 μg/L for cases and 45, 1.2, and 0–997 μg/L for controls, when proxy wells were excluded. Risks were not reported by sex.

Heck et al. (2009) studied 223 incident lung cancer cases from state cancer registries and 283 controls from a commercial database, frequency matched by five-year-age group and sex, in 10 counties in New Hampshire and Vermont. Participants were interviewed in person and were asked to provide biological samples (blood, toenail clippings, oral buccal cells from brushing, and oral cells from a mouthwash sample). Arsenic exposure was based on toenail clippings. Small-cell and squamous-cell carcinoma of the lung (OR = 2.75; 95% CI, 1.00–7.57) was associated with toenail arsenic concentration ≥ 0.114 μg/g versus < 0.05 μg/g. The authors also observed an elevated risk of lung cancer among participants with a history of lung disease and toenail arsenic ≥ 0.05 μg/g (OR = 4.78; 95% CI, 1.87–12.2) as compared with individuals with low toenail arsenic and no history of lung disease.

Karagas et al. (2004) reported 383 cases of transitional-cell carcinoma of the bladder identified from the New Hampshire Cancer Registry and 641 controls from the general population. Toenail arsenic was used as the measure of exposure. There was an increased risk of transitional cell bladder cancer among ever-smokers at the highest versus the lowest toenail arsenic concentration, but not among all subjects or never-smokers at any concentration. The geometric mean (GM) values of toenail arsenic [standard error (SE) of the GM] were 0.087 μg/g (SE = 0.003 μg/g) and 0.090 μg/g (SE = 0.002 μg/g) for cases and controls, respectively.

Meliker et al. (2010) studied 411 bladder cancer cases and 566 controls in 11 counties of southeastern Michigan (USA). Cases diagnosed between 2000 and 2004 were ascertained from the Michigan Cancer Surveillance program. Controls were frequency-matched to cases on age, sex, and race. Study participants were required to have lived in the defined area for at least 5 years. Water consumption, dietary habits, smoking, medical history, and residential and occupational history were determined by questionnaire. Lifetime arsenic exposure was determined based on residential history. No association between bladder cancer and arsenic concentration in drinking water was observed. Relative risks were not reported by sex.

Mostafa et al. (2008) published a study of 2,811 male lung cancer cases and 1,183 male controls in Bangladesh. The study compared primary lung cancer cases with cases with benign lung lesions. Arsenic exposure was determined through a sampling of tube wells by the British Geological Survey and responses to questionnaires on residence and tube well use. Among smokers, the relative risks (95% CIs), compared with the reference group (< 10 μg arsenic/L), were 1.25 (0.96–1.62), 1.37 (0.92–2.03), and 1.65 (1.25–2.18) for concentrations 11–50, 51–100, and 101–400 μg arsenic/L, respectively. Among nonsmokers, the researchers found neither an increasing trend in OR nor a statistically significant OR at any exposure level.

Michaud et al. (2004) studied 280 bladder cancer cases and 293 controls (all male) in Southwest Finland. Cases and controls were smokers at the time of enrollment into the Alpha-Tocopherol, Beta-Carotene Prevention Study. ORs were estimated from unconditional logistic regression models adjusted for age, date at toenail collection, trial intervention group (Alpha-Tocopherol or Beta-Carotene), number of cigarettes per day, and number of years of smoking. No relationships between toenail arsenic concentrations and bladder cancer risk were found. Median toenail arsenic concentrations were 0.110 μg/g (range = 0.014–2.62 μg/g) and 0.105 μg/g (range = 0.017–17.5 μg/g) for cases and controls, respectively.

Steinmaus et al. (2003) studied 181 primary bladder cancer cases and 328 controls in California and Nevada. Cases diagnosed between 1994 and 2000 were identified from the Nevada Cancer Registry and the Cancer Registry of Central California. Population controls were age and sex matched. Arsenic measurements for all community-supplied drinking water wells were obtained from the health departments of the respective states. Questionnaires were administered to all participants. Arsenic exposures were classified by the highest 1-year average, highest 5-year average, highest 20-year average, and cumulative exposure. The percentages of cases and controls consuming different concentrations of arsenic (0–19, 20–79, 80–120, and > 120 μg/L) were similar, with the vast majority consuming 0–19 μg/L. The only significant associations were found after a 40-year lag in ever-smokers exposed to > 80 μg arsenic/day (OR = 3.67; 95% CI, 1.43– 9.42). Relative risks were not reported by sex.

### Ecologic studies

Han et al. (2009) compared cancer incidence by the arithmetic mean arsenic concentrations of ground water in 44 counties in Idaho, USA. Arsenic concentrations for the counties were based on approximately 1,990 groundwater sources sampled between 1991 and 2005. Newly diagnosed cases of cancer of the bladder, kidney and renal pelvis, liver and bile duct, lung and bronchus, and non-Hodgkin lymphoma from 1991 to 2005 were identified from the Cancer Registry of Idaho. Counties were grouped into arsenic water concentrations of < 2 μg/L (*n* = 23), 2 to < 10 μg/L (*n* = 16), and ≥ 10 μg/L (*n* = 5). Using regression analysis and controlling for smoking, race, sex, body mass index, and population density, the researchers found no evidence of an association between arsenic and cancer incidence in the 44 counties.

Lamm et al. (2004) studied 133 U.S. counties that depend exclusively on groundwater. Bladder cancer standardized mortality ratios (SMR) for white males, from 1950 to 1979, were plotted against median county arsenic concentrations in groundwater. SMRs for white males were not related to the median or mean county arsenic concentrations. Median county arsenic concentrations ranged from 3 to 60 μg/L. Eighty-two percent of the population studied was assumed to have consumed 3–5 μg arsenic/L.

Meliker et al. (2007) investigated six counties in southeastern Michigan. Deaths for the six counties and for the state of Michigan from 1979 to 1997 were used to estimate the sex-specific SMRs. Data from Michigan Department of Environmental Quality from 1983 through 2002 were used to estimate county-level mean and median arsenic concentrations for the six counties and for the rest of Michigan. The SMRs for lung, bronchus, and trachea cancer and for bladder cancer were not significantly increased among the six counties as a whole. The SMRs for lung, trachea, and bronchus cancer were significantly increased in both males and females in Genesee County, the most populous, urban, and racially diverse of the six counties. The six-county study area had a population-weighted mean arsenic concentration of 11.00 μg/L and a population-weighted median of 7.58 μg/L. The population-weighted mean in the remainder of Michigan was 2.98 μg/L with a median of 1.27 μg/L.

## Discussion

As indicated by Table 4, few studies reported significantly increased risks of lung or bladder cancer from exposure to < 100 μg arsenic/L. That does not suggest, however, that the lung and bladder cancer excess risks estimated by the NRC are incorrect. The sample sizes, with type 1 error = 0.05 and power = 0.80, needed to detect the NRC-estimated excess risks of bladder cancer in females are relatively large (Table 3). The sample sizes needed to detect associations with lung cancer in females or lung or bladder cancer in males would be even larger. None of the studies conducted since the NRC report had sample sizes large enough to detect the excess risks estimated by the NRC. Nevertheless, there is evidence of increased lung and bladder cancer risk from exposures < 100 μg arsenic/L in the recent studies, particularly in the studies by Chen et al. (2010a, 2010b). The monotonic dose response seen across all exposures in Chen et al. (2010b) and the significant trend (*p* < 0.001) for dose response provides strong evidence for the bladder cancer risks seen at the lower exposures.

The three case–control studies that examined the relative risks of bladder cancer from low-arsenic drinking water concentration drew cases and controls from arsenic-endemic areas (Bates et al. 2004; Meliker et al. 2010; Steinmaus et al. 2003). The reason for drawing cases and controls from the same area is, of course, to minimize potential differences other than the factor under study (i.e., arsenic). Drawing cases and controls from the same area, however, may also reduce the difference in arsenic exposure, requiring a larger sample size to determine whether an excess risk exists for a given exposure. Exposure misclassification probably further reduced the difference between groups. Because the estimated exposure difference between cases and controls was minimal in each of these studies, the statistical power to detect the excess risks predicted by the NRC would also have been minimal.

Two of the case–control studies that examined bladder cancer (Karagas et al. 2004; Michaud et al. 2004) used toenail arsenic as the measure of exposure. According to Karagas et al. (2000), 1 μg arsenic/L water corresponds to 0.1 μg arsenic/g toenail, whereas a doubling of toenail arsenic concentration is associated with a 10-fold increase in water arsenic in samples with ≥ 1 μg arsenic/L. Based on this relationship, cases and controls in all three studies would have been exposed to approximately ≤ 1 μg arsenic/L. Even if this level of exposure were assumed to be lifetime, the power of the studies of Karagas et al. (383 cases, 641 controls) and Michaud et al. (280 cases, 293 controls) to detect the bladder cancer relative risk estimated by the NRC (2001) would have been minimal. Toenail arsenic is considered a reliable indicator of arsenic exposure, with the strongest relationship being drinking water–arsenic exposure (Adair et al. 2006). When the concentration of arsenic in water is low, however, the contribution of arsenic to toenail arsenic concentrations becomes less clear as other sources (e.g., food, air, dermal absorption) become more important (Slotnick and Nriagu 2006).

Han et al. (2009), Lamm et al. (2004), and Meliker et al. (2007) were ecologic studies. The exposure metric in all three was an average exposure measurement by county, a much larger geopolitical unit than the villages in the Taiwan study. Lamm et al. (2004) relied on U.S. Geological Survey (USGS) data for mean and median arsenic concentrations by county (Focazio et al. 1999). The USGS data were based on as few as five wells per county and were not restricted to wells used for drinking water. All three ecologic studies were conducted in the United States, where the population is very transient—much more transient than, for example, the southwest Taiwanese population that is the basis of the NRC risk estimates. Hansen (1998) reported that the median duration in a residence for those in the United States is only 5.2 years. In addition, the U.S. population consumes different sources of fluids (e.g., tap water from other jurisdictions, bottled beverages) in addition to potentially contaminated well water, whereas those in southwest Taiwan likely consumed well water as their principal, if not the only, source of fluids. Furthermore, both Lamm et al. (2004) and Meliker et al. (2007) examined bladder cancer mortality rather than incidence. The NRC estimates are for cancer incidence. Because the 5-year survival rate for bladder cancer is approximately 80% (American Cancer Society 2010), studies based on bladder cancer mortality would underestimate the risks described by NRC. Finally, the arsenic exposures in all three studies were very low (< 10 μg/L). Han et al. (2009) compared incidence of cancer for different sites by low (< 2 μg/L), medium (2–9 μg/L), and high (≥ 10 μg/L) arsenic counties. The median arsenic concentrations in the counties studied by Lamm et al. (2004) were 3–60 μg/L, with 65% of the counties and 82% of the population in the range of 3–5 μg/L. The six Michigan counties studied by Meliker et al. (2007) had population-weighted mean and median arsenic concentrations of 11.00 μg/L and 7.58 μg/L, respectively, compared with 2.98 μg/L and 1.27 μg/L for the remainder of the state. The differences were relatively small.

Some of the recent studies suggest that the ability of an epidemiologic study of arsenic exposure to detect associations with lung or bladder cancer could be impacted by the number of smokers in the study population. Bates et al. (2004), Karagas et al. (2004), and Steinmaus et al. (2003) all report an increased risk of bladder cancer in smokers, but not nonsmokers, exposed to relatively low concentrations of arsenic in drinking water. The ecologic study by Meliker et al. (2007) found a significant increase in lung cancer mortality for the most urbanized of the six arsenic-endemic counties (Genesee County). Because the prevalence of smoking in the most urbanized county was expected to be higher than in the other five counties, the authors suggested the significant elevation in lung cancer mortality is due to the synergy between arsenic and smoking. An earlier study by Kurttio et al. (1999) also presents evidence of an interaction between smoking and arsenic with respect to bladder cancer, while Chen et al. (2004), Ferreccio et al. (2000), and Mostafa et al. (2008) suggest an interaction between smoking and arsenic with respect to lung cancer. Chen et al. (2010a) found a synergistic effect of arsenic exposure and cigarette smoking for squamous- and small-cell carcinomas of the lung but not for adenocarcinoma. The authors also reported that a relationship between smoking, arsenic, and lung cancer was evident by the significantly elevated RRs among exposed smokers compared with exposed nonsmokers.

The study by Chen et al. (2010b) found that drinking arsenic-contaminated water since birth had a higher urinary cancer risk than beginning to drink arsenic-contaminated water later in life. Smith et al. (2006), in a study of lung cancer risk in an arsenic-endemic area where drinking water concentrations were relatively high, found that early lifetime exposure may convey a greater risk for lung cancer. Bates et al. (1995, 2004), Marshall et al. (2007), and Steinmaus et al. (2003) found that the latency for arsenic-induced cancer was particularly long, indicating that a 40- to 50-year follow-up may be required to detect an excess risk.

The recent studies by Chen et al. (2010a, 2010b) are noteworthy for their potential to improve the quantitative risk assessment for arsenic. The population is relatively large (8,086) and includes a large number exposed since birth. Arsenic-contaminated drinking water concentrations range from < 10 μg/L to > 300 μg/L. Individual drinking water measurements are available for most in the cohort, as opposed to the village measurements on which the NRC risk estimates are based. Data are also available on age of the individuals, sex, education, cigarette smoking, habitual alcohol consumption, age when the individuals started drinking arsenic-contaminated well water, and age when the individual stopped drinking arsenic-contaminated well water. Additional follow-up of this cohort will provide a valuable database for future risk assessments. Individual data from the study will be needed to do a quantitative risk assessment. The Health Effects of Arsenic Longitudinal Study (HEALS) of an arsenic-exposed cohort of almost 20,000 in Bangladesh (Ahsan et al. 2006; Chen et al. 2009) may also be of use in the future in assessing cancer risk at low arsenic exposures. The study has collected individual data on smoking, education, socioeconomic status, skin lesions, arsenic exposure (including biomarkers of exposure), and other variables.

## Conclusion

The NRC estimated excess lifetime lung and bladder cancer risks based on a lifetime of exposure to arsenic in drinking water. Since 2001, several studies have evaluated lung and bladder cancer risk in persons consuming low concentrations of arsenic in drinking water (≤ 100 μg/L). These studies lack either the statistical power or the information necessary to evaluate the bladder and lung cancer risk estimates noted in the NRC report (2001). Validating the NRC-estimated risks is problematic given the sample sizes needed, the long latency involved, and the greater need to control for confounders at low-arsenic drinking water concentrations. Some of the recent studies, however, suggest that lung and bladder cancer risks are increased at concentrations ≤ 100 μg arsenic/L. Future studies on arsenic ingestion and lung and bladder cancer risk would benefit by considering the adequacy of the sample size, the synergistic relationship of arsenic and smoking, duration of arsenic exposure, age when exposure began and ended, and examination by histological type. Although the data from southwest Taiwan continue to provide the best basis for the quantitative risk assessment of lung and bladder cancer from ingested arsenic, individual data from the continued follow-up of the population studied by Chen et al. (2010a, 2010b) should provide an excellent database on which to improve the assessment.

## Figures and Tables

**Table 1:** NRC (2001)^a^ estimates of lifetime cancer risks per 10,000 people in relation to arsenic concentration.

Concentration (μg arsenic/L)	Cancer risk			
	Lung		Bladder	
	Females	Males	Females	Males
3	5	4	4	7
5	9	7	6	11
10	18	14	12	23
20	36	27	24	45

a Reprinted with permission the National Academies Press (2001).

**Table 2:** Excess lifetime risks, background lifetime risks, and relative lifetime risks for bladder and lung cancer from a lifetime consumption of 10 μg arsenic/L, by sex.

Type of cancer	NRC-excess lifetime risk^a^	Background- lifetime risk^b^	NRC-relative lifetime risk^c^
Bladder			
Males	0.0023	0.0315	1.07
Females	0.0012	0.0094	1.13
Lung			
Males	0.0014	0.0689	1.02
Females	0.0018	0.0554	1.03

Lifetime risk is through age 85 years.

a Data from NRC (2001).

b Data from SEER (2006a, 2006b).

c Relative lifetime risk = (excess lifetime risk + background lifetime risk) ÷ background lifetime risk.

**Table 3:** Sample sizes required for 80% statistical power and type 1 error = 0.05 to detect bladder cancer in a cohort study of females for a range of arsenic concentrations.

Arsenic (μg/L)	Excess risk^a^	RR^b^	Sample size (80% power)
100	0.012	2.28	672
50	0.006	1.64	2,381
20	0.0024	1.26	13,613
10^c^	0.0012	1.13	52,640
3	0.0004	1.04	462,527

a Estimates from NRC (2001).

b Background [(excess lifetime risk + background lifetime risk) ÷ background lifetime risk. The lifetime risk for women up to age 85 years for bladder cancer, years 2004–2006, is 0.0094 (0.94%) (NCI 2010).

c Current U.S. EPA MCL for arsenic is 10 μg/L (U.S. EPA 2001c).

**Table 4:** Studies published since 2001 that examined the risk of lung, urinary, and bladder cancer for arsenic concentrations in drinking water < 100 μg/L.

Authors	Study size	Location	Cancer type	Measures of association (95% CI)
Cohort studies				

Baastrup et al. 2008	56,378	Denmark	Bladder	0.7 μg/L [IRR = 1.01 (0.93–1.11)]
			Lung	0.7 μg/L [IRR = 0.99 (0.92–1.07)]

Chen et al. 2004	2,503 8,088	Southwest Taiwan Northeast Taiwan	Lung	< 10 μg/L; RR = 1.0
				10–99 μg/L [RR = 1.09 (0.63–1.91)]
				100–299 μg/L [RR = 2.28 (1.22–4.27)]
				300–699 μg/L [RR = 3.03 (1.62–5.69)]
				≥ 700 μg/L [RR = 3.29 (1.60–6.78)]
				*p*-trend < 0.001

Chen et al. 2010a	8,086	Northeast Taiwan	Lung	< 10 μg/L; RR = 1.0
				10–49.9 μg/L [RR = 1.10 (0.74–1.63)]
				50–99.9 μg/L [RR = 0.99 (0.59–1.68)]
				100–299 μg/L [RR = 1.54 (0.97–2.46)]
				≥ 300 μg/L [RR = 2.25 (1.43–3.55)]
				*p*-trend = 0.001

Chen et al. 2010b	8,086	Northeast Taiwan	Urinary	< 10 μg/L; RR = 1.0
				10–49.9 μg/L [RR = 1.66 (0.53–5.21)]
				50–99.9 μg/L [RR = 2.42 (0.69–8.54)]
				100–299 μg/L [RR = 4.13 (1.32–12.9)]
				≥ 300 μg/L [RR = 7.80 (2.64–23.1)]
				*p*-trend < 0.001

Case–control studies				

Bates et al. 2004	114 cases 114 controls	Argentina	Bladder	For arsenic exposure 1–10 years before interview including proxy well measurements:
				0–10 μg/L; OR = 1.00
				> 10 μg/L [OR = 0.75 (0.4–1.7)]

Heck et al. 2009	223 cases 283 controls	New Hampshire and Vermont, USA	Lung	NA—exposure measured by toenail arsenic

Karagas et al. 2004	383 cases 641 controls	New Hampshire, USA	Bladder	NA—exposure measured by toenail arsenic

Meliker et al. 2010	411 cases 566 controls	Southeastern Michigan, USA	Bladder	< 1 μg/L; OR = 1.00
				1–10 μg/L [OR = 0.84 (0.63–1.12)]
				> 10 μg/L [OR = 1.10 (0.65–1.86)]

Michaud et al. 2004	280 cases 293 controls	Southwest Finland	Bladder	NA—exposure measured by toenail arsenic

Mostafa et al. 2008	2,811 cases 1,183 controls	Bangladesh	Lung	Smokers
				≤ 10 μg/L; OR = 1.0
				11 ≤ 50 μg/L [OR = 1.25 (0.96–1.62)]
				51 ≤ 100 μg/L [OR = 1.37 (0.92–2.03)]
				101–400 μg/L [OR = 1.65 (1.25–2.18)]
				Nonsmokers
				≤ 10 μg/L; OR = 1.0
				11 ≤ 50 μg/L [OR = 0.90 (0.62–1.33)]
				51 ≤ 100μg/L [OR = 1.10 (0.62–1.96)]
				101–400 μg/L [OR = 0.94 (0.62–1.41)]

Steinmaus et al. 2003	181 cases 328 controls	California and Nevada, USA	Bladder	For arsenic exposures with a 40-year lag and highest 20-year average:
				< 10 μg arsenic/day; OR = 1.0
				10–80 μg arsenic/day [OR = 1.28 (0.53–3.11)]
				> 80 μg arsenic/day [OR = 1.70 (0.73–3.96)]

Ecologic studies				

Han et al. 2009	44 counties	Idaho, USA	Bladder	Pearson correlation coefficient of arsenic in ground water and bladder cancer incidence = 0.02 (*p* = 0.9)
			Lung	Pearson correlation coefficient of arsenic in ground water and lung cancer incidence = 0.25 (*p* = 0.1)

Lamm et al. 2004	133 counties	USA	Bladder	3–60 μg/L (82%; 3–5 μg/L):
				Regression analysis found no association of bladder cancer mortality with arsenic in drinking water

Meliker et al. 2007	6 counties	Southeastern Michigan, USA	Bladder	7.58 μg/L (population-weighted median) [SMR = 0.94 (0.82–1.08)]
			Lung	7.58 μg/L (population-weighted median) [SMR = 1.02 (0.98–1.06)]
